# Laminated pyroelectric generator with spin coated transparent poly(3,4-ethylenedioxythiophene) polystyrene sulfonate (PEDOT:PSS) electrodes for a flexible self-powered stimulator†

**DOI:** 10.1039/c8ra00491a

**Published:** 2018-04-23

**Authors:** Weitao Jiang, Tingting Zhao, Hongzhong Liu, Rui Jia, Dong Niu, Bangdao Chen, Yongsheng Shi, Lei Yin, Bingheng Lu

**Affiliations:** State Key Laboratory for Manufacturing Systems Engineering, Xi'an Jiaotong University Xi'an 710049 China wtjiang@mail.xjtu.edu.cn hzliu@mail.xjtu.edu.cn; Department of Neurology, First Affiliated Hospital of Xi'an Jiaotong University Xi'an 710061 China

## Abstract

Implantable devices are promising electronics in medicine, which can perform real-time monitoring for a variety of human-body physiological conditions and control the function of some failing organs. However, the technology to power implantable devices still has some remaining challenges. This work presents a transparent self-powered pyroelectric generator driven by near infrared radiation for wireless powering of electronics. The pyroelectric device uses a highly conductive polymer, PEDOT:PSS, formed as an electrode without the use of a complex transferring process. Due to the good match between the surface energy of the PEDOT electrode and PVDF, when combined with PVDF the resulting PEDOT/PVDF/PEDOT device possesses a highly adherent interface. The influence of the PEDOT thickness on the output voltage of the device has been investigated according to the difference in its infrared transmittance and absorbance. In addition, in order to enhance the output voltage while reducing the device temperature, a laminated pyroelectric generator, in which each cell is composed of a PEDOT/PVDF/PEDOT sandwich, was further developed taking advantage of the high infrared transmittance of PEDOT and PVDF. The proposed laminated pyroelectric device could generate up to 23.4 V with six laminated cells, an enhancement of approximately 212% compared to a single cell, which could directly light up an LCD and was applied for nerve stimulation of the sciatic nerve of a frog, indicating that the proposed self-powered device could be a candidate for implantable medical electronics.

## Abstract 2

A laminated pyroelectric generator with spray coated transparent PEDOT:PSS electrodes has been designed for use as a flexible self-powered stimulator.

## Graphical abstract

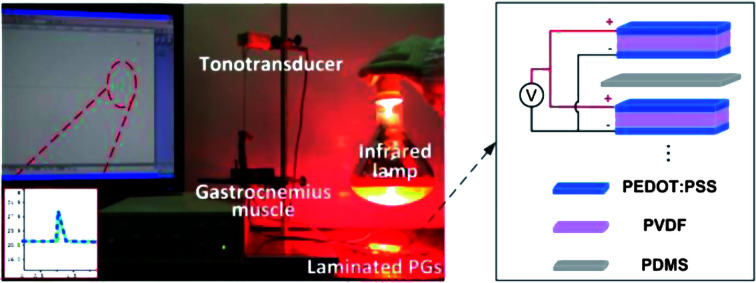

## Introduction

In recent years, the development of medical science and electronic technology has made implantable medical devices an important topic in medicine.^1,2^ They can not only perform *in situ* and real-time monitoring for different human-body physiological conditions but can also control the function of failing organs by sending an electrical pulse to a major nerve to alter the commands an organ receives.^3,4^ Due to their excellent performance, that is more precise than conventional pharmaceuticals, more implantable medical devices, such as pacemakers, drug pumps, cochlear implants, neurological stimulators and so on, are widely used in medicine.^5,6^ Currently, nearly all implantable medical devices rely on some form of battery power for normal operation in the body. However, due to the limited lifespan of batteries, increased efforts have been dedicated to looking for a sustainable power supply, in order to reduce the health risks to patients and the cost brought about by surgical procedures that are needed to replace depleted batteries.^7^ Meanwhile, an integrated power supply can occupy >90% of the implantable device volume, which results in a high demand for miniaturized power supplies.^8^ Besides these factors, in order to have a minimal inflammatory response and to be stable in a body over a long time period, the mechanical flexibility of a power supply is highly desirable, which determines whether a conformal contact with tissue can be formed.^9^ Therefore, the lifespan, size and flexibility of an implantable power supply remain the greatest challenges.

An attractive approach to address these challenges is to design a self-powered battery that uses energy harvesting technology, which can convert energy from natural processes of the body into electricity to power implantable medical devices. Recent examples involve the use of chemical energy from glucose oxidation,^10^ and gastric acid,^11^ or mechanical energy from respiration,^12^ natural vibrations of some organs, such as the heart,^13^ ascending aorta^14^ and diaphragm,^7^ and so on. Although such means provide opportunities for powering implantable devices, the power densities of these systems are generally quite low, and the controllability is relatively weak due to the limitation of the fixed *in vivo* environment.^8^ Alternatively, a number of approaches for self-powering systems that require the scavenging of external energy from environments by wireless transport in order to power implants have been explored. Representative examples mainly include the use of electromagnetic coupling and ultrasonic waves.^15,16^ Although electromagnetic coupling can provide high power density for implants, its energy transfer distance, miniaturization and flexibility remain key challenges.^8^ Wireless power through ultrasonic waves has the advantage of a long energy transmission distance, but the transferred power may fall to a minimum at a given distance due to the standing wave effect, resulting in low efficiency.^16^ Compared to the above energy sources, near infrared radiation (nIR, 760–1500 nm in wavelength), known for its photothermal effect and strong penetration ability and can penetrate into human tissue by approximately 4–10 cm,^17^ has been widely used in medical physical therapy.^18,19^ A nIR-driven pyroelectric generator (PG) can provide alternative wireless power for implants.^20^ In addition, the pyroelectric effect can convert temperature fluctuations into electricity and does not require any moving parts, thus enhancing the lifetime of the device compared to those of piezoelectric/triboelectric devices.

A pyroelectric device generally consists of a pyroelectric material and two electrode layers. Polyvinylidene difluoride (PVDF) is a kind of pyroelectric polymer material, exhibiting the characteristics of being lightweight, with mechanical flexibility and biocompatibility, which has attracted attention for use in implantable devices.^21–23^ In fact, some nanogenerators using PVDF have already been demonstrated for powering implants based on human-activities, such as breathing,^24^ the pulsation of an ascending aorta,^25^*etc.* To extract the generated charge, the PVDF film is coated with thin electrode layers. Traditional electrode materials, such as metals and indium tin oxide (ITO), can be easily fabricated and have excellent electrical conductivity, but the brittleness of these materials limits their application.^26^ For this reason, recent work in this area has focused on exploring novel materials with remarkable flexibility and conductivity, such as graphene^27^ and carbon nanotubes (CNTs).^28^ However, the poor adhesion of these electrodes on PVDF may cause a poor interfacial contact between the layers, resulting in low fatigue properties. The next generation of implantable electronics will require a conductive, flexible and highly adherent electrode material. To overcome this problem, some researchers have attempted to use conductive polymers as an alternate electrode material in electronics.^29,30^ Poly(3,4-ethylenedioxythiphene)/poly(4-styrenesulfonate) (PEDOT:PSS) is one of the most promising commercially available conductive polymers. It exhibits high transparency,^30^ conductivity,^31^ has a simple fabrication process, and has been applied in enhancing the photo-conversion efficiency of solar cells with some success.^32^

In this paper, we propose a nIR-driven pyroelectric generator composed of PVDF and PEDOT:PSS for the wireless powering of implants. When near infrared radiation remotely illuminates the device periodically, the pyroelectric device absorbs the heat provided by nIR and generates an electric pulse. The influence of the PEDOT thickness on the output voltage of devices has been investigated according to the difference in its infrared transmittance and absorbance. Moreover, in order to enhance the output voltage while reducing the device temperature, a laminated pyroelectric generator, in which each cell is composed of a PEDOT/PVDF/PEDOT sandwich, has been developed by taking advantage of the high infrared transmittance of PEDOT and PVDF. The laminated pyroelectric generator generates a voltage of up to 23.4 V with six laminated cells, an obvious enhancement of about 212% compared to a single cell, with a temperature rise of only 7 K. The generated electrical energy can illuminate a LCD and be applied to the nerve stimulation of the sciatic nerve of a frog, indicating that the proposed device can find broad application prospects as a novel power source for implantable electronics in the future.

## Experiment section

### Fabrication and characterization of the pyroelectric generator (PG)

PVDF film with a thickness of 30 μm was purchased from Jinzhou KeXin electronic materials Co. Ltd. The pyroelectric coefficient of the PVDF film is 40 μC m^−2^ K^−1^. PEDOT:PSS was used as the electrode material, provided by the Wuhan Zhuoxin science and technology Co. Ltd., and was deposited on the PVDF film using a spin-coating method. The rotation speed of the spin coater determined the thickness of PEDOT:PSS, which was measured by atomic force microscopy (AFM) (Innova, Veeco Instruments Inc.). In order to evaluate the electrode adhesion to PVDF, different eletrodes on PVDF, such as graphene and multi-walled carbon nanotube (MWCNT), were also prepared. The laminated device was fabricated by laminating many PG cells in parallel separated by transparent thin PDMS film layers (of about 1 μm in thickness). All of the positive and negative terminals of each PG cell were connected, respectively. An infrared laser (*λ* = 808 nm, *S*_light spot_ = 5 mm × 2 mm) was employed. Infrared light was periodically used to illuminate the top of the device using a controlled shutter to offer controllable irradiation. The temperature of the PG was measured using a thermal infrared imager (SC7300M, FLIR Systems AB). The electrical properties of the PG were measured using a digital phosphor oscilloscope (Tektronix DPO 3034) and a semiconductor characterization system (KEITHLEY 4200).

### Real-time functional electrical stimulation (FES) experiments

All animal experiments were performed in accordance with the Xi'an Jiaotong University Health Science Center and the First Affiliated Hospital of Xi'an Jiaotong University. The positive and negative electrode terminals of the laminated pyroelectric device were connected to the screw bolts of a stimulating electrode attached to the sciatic nerve of an interceptive hind limb of a frog. Muscle contraction was observed according to the tension response through a tonotransducer. In the experiments, the nerve and gastrocnemius muscle were kept wet using Ringers solution to ensure biological activity. The nerve stimulation experiments were generally performed ten minutes after dissection. In this study, all animal procedures were performed in conformity with the National Institutes of Health (NIH) guidelines for the care and use of laboratory animals (NIH Publication no. 85-23, Rev. 1985), and were approved by the Animal Care and Use Committee of Xi'an Jiaotong University (Xi'an, China). All efforts were made to minimize the number of animals used, as well as the distress caused to the animals.

## Results and discussion

**Fig. 1:**
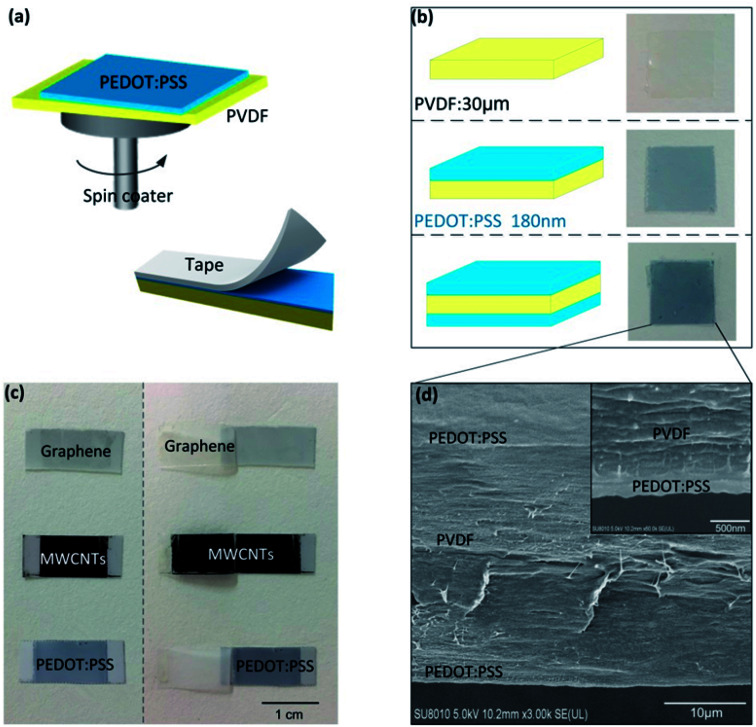
(a) A schematic diagram of the spin-coating method used for fabricating the PEDOT:PSS electrode layer and the taping test to verify the adhesion properties and stability of the electrodes. (b) Schematic diagrams and photographs of the PVDF film, PEDOT/PVDF, and PEDOT/PVDF/PEDOT. (c) A photograph of the adhesion test of different electrode materials and PVDF. (d) Cross-sectional SEM images of the interface between the PEDOT:PSS electrode and PVDF.

The pyroelectric generator has PVDF film and electrode materials on both sides. A PEDOT:PSS layer was coated onto the surface of the PVDF film (20 mm × 20 mm) using a spin coater, as shown in Fig. 1a. Fig. 1b shows schematic diagrams and photographs of the bare 30 μm PVDF film, the PVDF film covered by PEDOT:PSS on one side and the PVDF film covered by PEDOT:PSS on both sides, respectively. Here, the thickness of the PEDOT:PSS was about 180 nm with a spin-coating speed of 2400 rpm. A tape detachment test was undertaken to verify the adhesion characteristics of different electrode materials, as illustrated in Fig. 1a. The 3M tape was firstly stuck onto the electrode layer of the PVDF films and pressed repeatedly using 500 g weights, and was then detached from the electrode layer with almost the same speed. While graphene and most of the MWCNT films detached from the PVDF surface, the PEDOT:PSS film was well adhered to the PVDF surface, as shown in Fig. 1c. Even after several repeated detachment tests, the PEDOT:PSS layer was still retained on the surface of the PVDF film, which proved the good adhesion characteristics between PEDOT:PSS and PVDF. Through cross-section scanning electron microscopy (SEM) imaging of the PEDOT/PVDF/PEDOT (Fig. 1d), the interface between the PVDF and PEDOT:PSS could be seen to make tight contact without any distinct interfacial gap, which also confirmed the good adhesion characteristics between PEDOT:PSS and PVDF.

**Fig. 2:**
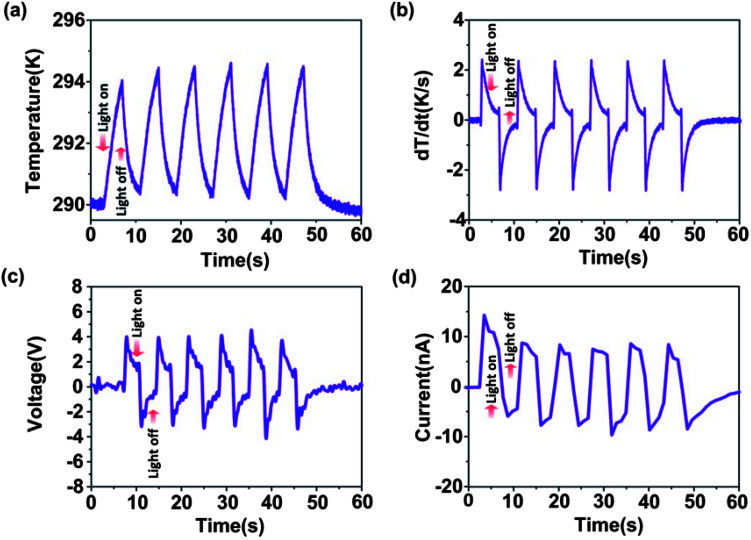
The (a) temperature, (b) temperature-change rate, (c) output voltage, and (d) short-circuit current for the pyroelectric generator with PEDTO:PSS as an electrode at an irradiation frequency of 0.125 Hz.

Fig. 2 shows the temperature profile, temperature-change rate, output voltage, and short-circuit current of the pyroelectric generator with PEDOT:PSS as an electrode at an irradiation frequency of 0.125 Hz (the irradiation on/off time is 4 s/4 s). The light intensity of the near infrared radiation is 1.45 W cm^−2^. When the near infrared radiation illuminated the PG, a temperature rise from 290 K to 294 K was observed, with a sharp positive voltage of 4 V and short-circuit current of 15 nA at a temperature-change rate of 2.5 K s^−1^. When the near infrared radiation was turned off, a temperature drop from 294 K to 290.5 K was observed, with a negative voltage/current pulse response (∼3.5 V; −5 nA). According to pyroelectric theory,^33^ the short circuit pyroelectric current *i* can be given by:1
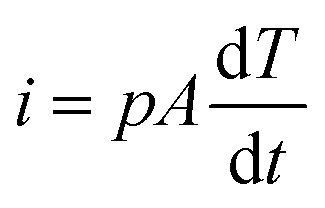
where *p* is the pyroelectric coefficient (units: C m^−2^ K^−1^), *A* is the electrode area (units: m^2^), and d*T*/d*t* is the temperature-change rate (units: K s^−1^). According to eqn (1), when the temperature-change rate of the PG increases, a positive pyroelectric voltage/current signal should be observed under the forward connection to the measurement system due to the positive pyroelectric coefficient, which is consistent with the experimental data shown in Fig. 2. Both the pyroelectric current and voltage can be enhanced by increasing the temperature-change rate of the PG with fixed pyroelectric coefficient and electrode area.

**Fig. 3:**
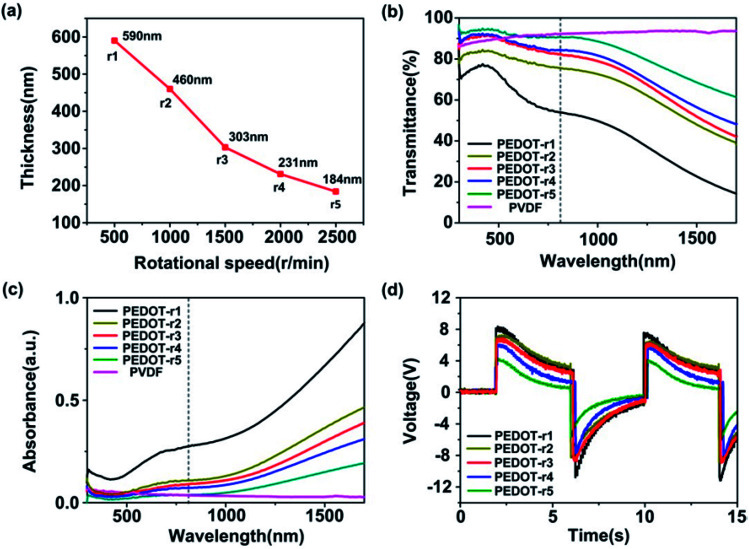
(a) Measured PEDOT thickness under different rotating speeds (*r*_1_ = 600 rpm, *r*_2_ = 1200 rpm, *r*_3_ = 1500 rpm, *r*_4_ = 2000 rpm, *r*_5_ = 2500 rpm). (b) The transmittance spectra and (c) the absorbance spectra of the PVDF film and PEDOT:PSS film under different rotation speeds. (d) Measured output voltages of PG (PEDOT/PVDF/PEDOT) with different PEDOT thicknesses.

The thickness of PEDOT can influence its infrared transmittance and absorbance, which affects the temperature of the PG, further determining the output electricity, according to eqn (1). We measured the infrared transmittance and absorbance of PEDOT films with different thickness determined by the rotating speed of the spin coater. When the rotation speed increased from 600 rpm to 2500 rpm, the PEDOT thickness decreased from 590 nm to 184 nm, as shown in Fig. 3a (*r*_1_ = 600 rpm, *r*_2_ = 1200 rpm, *r*_3_ = 1500 rpm, *r*_4_ = 2000 rpm, *r*_5_ = 2500 rpm). The AFM data of the PEDOT thickness for various rotation speeds are shown in Table S1 in the ESI.† According to the results in Fig. 3b and c, it could be found that the infrared transmittance of PEDOT decreased, while its infrared absorbance increased upon an increase in the thickness of PEDOT:PSS. For near infrared radiation with a wavelength of 808 nm, the transmittance of the PEDOT film could be up to 89% at a rotation speed of 2500 rpm. In contrast, its absorbance reduced to almost zero. The output voltages of PEDOT/PVDF/PEDOT with different PEDOT thicknesses were measured and the results are shown in Fig. 3d. We found that the higher the rotation speed, the lower the output voltage from the PG. This was because a higher rotation speed led to the higher infrared transmittance and lower infrared absorbance of PEDOT, and most of the infrared radiation penetrated through the PG and could not be absorbed, resulting in a lower temperature rise.

Although the output voltage of the PG device could be enhanced using thicker PEDOT, the temperature of the device also increased. However, temperature is one of the critical issues for implantable devices. A temperature change outside the safe range could bring about potential adverse biological responses. Some investigations about clinical hyperthermia and its biological effects in previous studies has indicated that the safe temperature range is 10–13 K for bones and 9 K for tissues, which are insufficient to induce irreversible damage.^34,35^ Therefore, in order to lower the temperature rise of the device, a PEDOT:PSS film made as thin as possible was preferably selected as an electrode material due to its low infrared absorbance. However, the output voltage of the device reduced correspondingly. According to the transmittance spectra shown in Fig. 3b, we found that the infrared transmittance of the PEDOT film under a rotation speed of 2500 rpm and PVDF with a thickness of 30 μm could be up to 91.2% and 92.5%. Based on this, it was possible to laminate several PEDOT/PVDF/PEDOT cells to ensure that more cells could absorb the nIR irradiation, thus increasing the size of the heating area. Therefore, in order to further improve the output power of the pyroelectric device, we connected several laminated pyroelectric devices in parallel utilizing the transparency of PEDOT and PVDF.

**Fig. 4:**
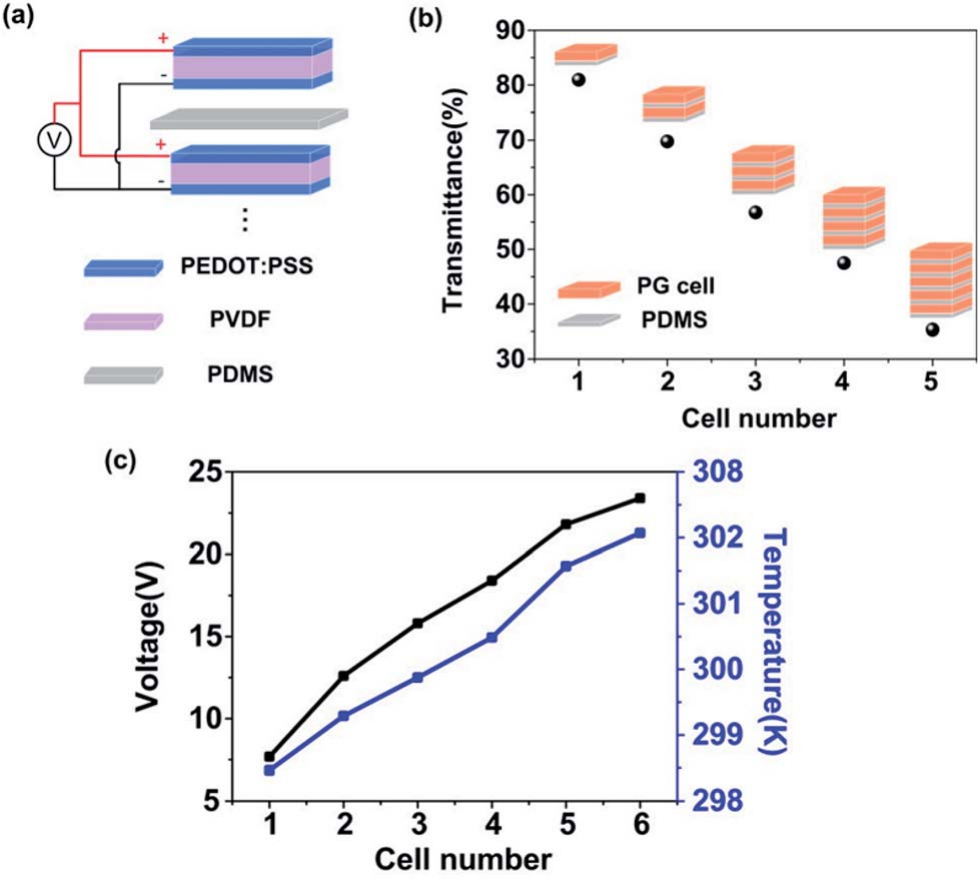
(a) A schematic diagram of the flexible laminated PG device. (b) The infrared light transmittance through the laminated device with different numbers of cells. (c) The output voltage and temperature of the laminated devices with different numbers of cells.

Fig. 4a shows a schematic diagram of the flexible laminated PG device. The laminated PG device was fabricated by laminating many PG cells with PEDOT electrodes on both sides of the PVDF film in parallel separated by some insulating layers. All of the positive and negative electrodes from each PG cell were connected respectively. Fig. 4b shows the transmittance of the nIR decreasing upon an increase in the number of PG cells. When the cell number reached five, the infrared transmittance reduced to 35%. This indicated that the intensity of the near infrared light gradually dropped from the top cell to the bottom. Therefore, the output voltage of every pyroelectric device cell will gradually drop from top to bottom. Fig. 4c shows the laminated cell number dependence of the output voltage and the temperature on the top of the device. The output voltage can be up to 23.4 V with six laminated PG cells, an obvious enhancement of about 212% compared to a single cell (7.5 V). It should also be noted that the highest temperature of the laminated device with six cells when irradiated is 302 K, only a 7 K increase to the base temperature (295 K), which is within the safe temperature range to avoid tissue damage. This low temperature rise is due to the weak infrared absorbance of PEDOT and PVDF, which is beneficial for implants. Mechanical fatigue and durability tests were also conducted (See Fig. S1 and S2 in the ESI†) to verify the performance of the laminated device, which barely deteriorated after over thousands of bending tests and after working for a long time.

**Fig. 5:**
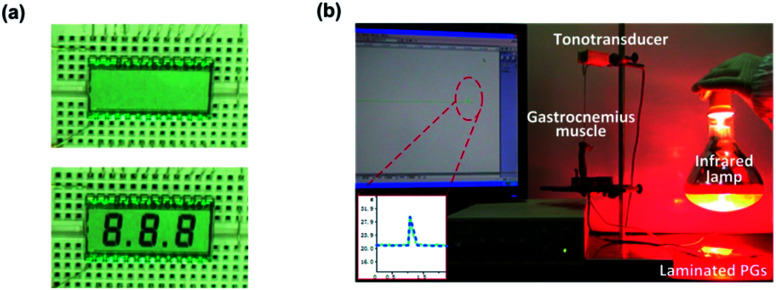
(a) A LCD screen operated by electricity generated from the laminated PG device without external circuits. (b) A labelled photograph of the experimental set-up for the electrical stimulation of the gastrocnemius muscle of a frog.

To illustrate the wireless powering ability of the laminated PG, a LCD screen was connected to the laminated PG without an external circuit. The output voltage of the laminated PG with six cells reached 2.3 V (with a load resistance of 10 MΩ), which was enough to light a commercial LCD. When the nIR irradiated the PG from a distance of two meters away, three characters appeared on the LCD screen. When the nIR was not applied to the PG, the characters disappeared, as shown in Fig. 5a. This was because the symmetry of the voltage waveform is dependent on the irradiation frequency.^28^ When the irradiation frequency was 1 s/4 s, the output negative voltage was low due to the short irradiation time, which was not enough to light the LCD. Therefore, the characters disappeared when the nIR left the PG. The wireless powering ability of the laminated PG driven by nIR is suitable for some special sets of circumstances, in which near-field control is not available, such as in implantable power supplies.

Then, we employed this laminated PG as an electrical stimulator for the real-time functional electrical stimulation of a gastrocnemius muscle of a frog by nIR irradiation. An image of the experimental set-up is shown in Fig. 5b. A specimen of the gastrocnemius muscle with a sciatic nerve was prepared and connected to a tonotransducer. For the gastrocnemius muscle of the frog, a contraction was produced when the stimulus voltage value was beyond the threshold voltage of its resting potential. In general, a voltage of 50 mV was essential to induce innervation.^36^ The output voltage of this laminated PG met the requirements for the electrical stimulation of the gastrocnemius muscle. When the nIR irradiated the laminated PG, the muscle contraction was clearly observed through a tension response, as shown in the inset in Fig. 5b. This result indicated that the nIR driven pyroelectric device would find potential application in the development of a self-powered electrical stimulator.

## Conclusion

A transparent wireless-powering pyroelectric generator driven by nIR was obtained using a highly conductive polymer, PEDOT:PSS, as an electrode without the use of a complex transferring process. Due to the good match between the surface energy of the PEDOT electrode and the PVDF, the resulting PEDOT/PVDF/PEDOT device possessed a highly adherent interface. When near infrared radiation remotely illuminated the device periodically, the pyroelectric device could absorb heat provided by nIR and generate an electric pulse. It was observed that the output voltage of the pyroelectric device was determined by the thickness of the fabricated PEDOT:PSS. The voltage was gradually enhanced as the PVDF thickness increased. This was because the increase in PEDOT thickness led to an elevated infrared absorbance and decreased infrared transmittance, resulting in the higher temperature rise of the device. In addition, in order to enhance its output voltage while reducing the device temperature, a laminated PG device, in which each cell was composed of a PEDOT/PVDF/PEDOT sandwich with the thinnest PEDOT layer, was further developed based on the high transparency of PEDOT and PVDF. The proposed laminated pyroelectric device could generate voltages of up to 23.4 V using six laminated PG cells, an obvious enhancement of about 212% compared to a single cell. The generated electric energy effectively powered a LCD, and directly realized the nerve stimulation of the sciatic nerve of a frog, indicating that the proposed self-powered device can find wide applications for use in implantable electronics.

## Conflicts of interest

There are no conflicts to declare.

## Supplementary Material

RA-008-C8RA00491A-s001
