# Supplementary material for: Laminated pyroelectric generator with spin coated transparent poly(3,4-ethylenedioxythiophene) polystyrene sulfonate (PEDOT:PSS) electrodes for a flexible self-powered stimulator
Source: RSC Adv. 2018 Apr 23;8(27):15134–40. doi: 10.1039/c8ra00491a (PMC9079999; doi:10.1039/c8ra00491a)
Supplement: RA-008-C8RA00491A-s001 [file RA-008-C8RA00491A-s001.pdf]

Electronic Supplementary Material (ESI) for RSC Advances.
This journal is © The Royal Society of Chemistry 2018

Laminated pyroelectric generator with spin coated transparent poly(3,4-ethylenedioxythiophene) polystyrene sulfonate (PEDOT:PSS) electrodes for flexible self-powered stimulator

Weitao Jiang[a,*], Tingting Zhao[a], Hongzhong Liu[a,*], Rui Jia[b], Dong Niu[a], Bangdao Chen[a], Yongsheng Shi[a], Lei Yin[a], Bingheng Lu[a]

[a]State Key Laboratory for Manufacturing Systems Engineering, Xi'an Jiaotong University, Xi'an 710049, China. [b]Department of Neurology, First Affiliated Hospital of Xi'an Jiaotong University, Xi'an 710061, China.
*Corresponding authors
E-mail: wtjiang@mail.xjtu.edu.cn, hzliu@mail.xjtu.edu.cn

Supporting information

Table S1. **The AFM data of PEDOT thickness under different rotation speeds**

	Sample 1	Sample 2	Sample 3	Sample 4	Mean value
500 r/min	588.817	590.361	591.580	589.290	590.012 nm
1000 r/min	458.263	461.390	460.720	461.087	460.365 nm
1500 r/min	303.580	301.234	304.380	301.298	302.623 nm
2000 r/min	235.638	230.520	229.541	230.109	231.452 nm
2500 r/min	188.246	185.012	182.538	181.008	184.201 nm

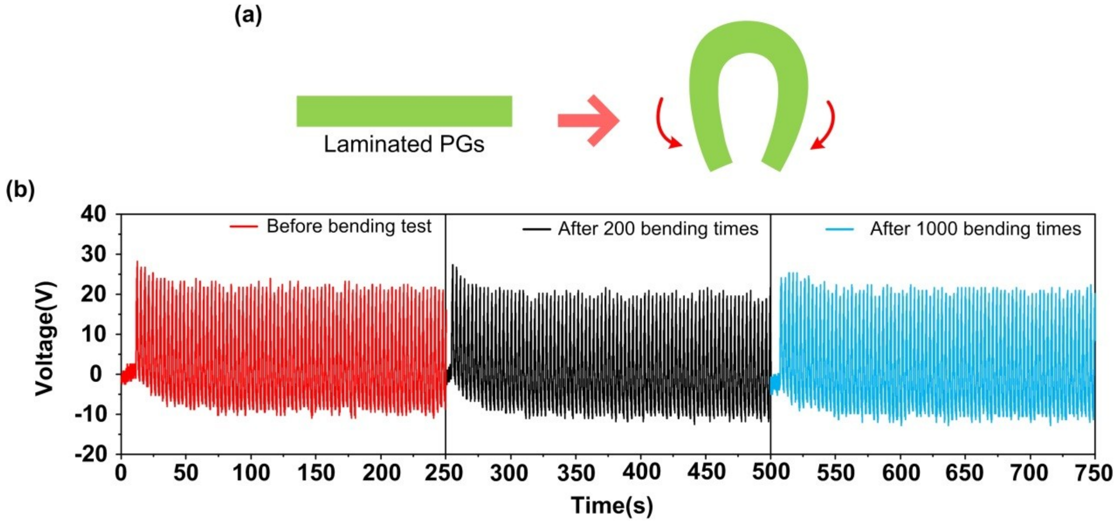

Figure S1. (a) Bending schematic diagram. (b)The voltage of laminated device was measured before mechanical fatigue tests and after 200, 1000 bending times.

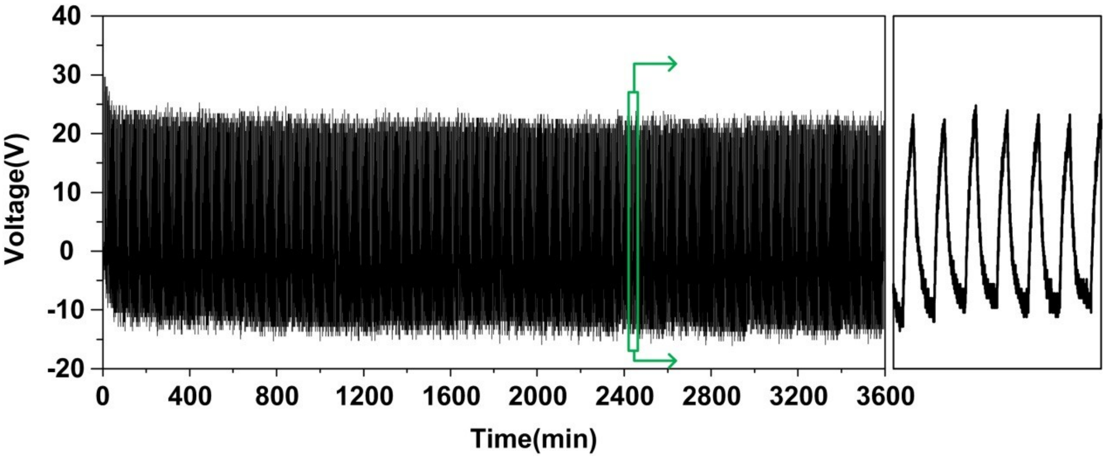

Figure S2. The electrical output of a laminated device during 1 h when irradiation on and off time is kept at 1s/2s. The 1 h of continuously working of the device demonstrates its stability and durability.
